# Role of the Platelets and Nitric Oxide Biotransformation in Ischemic Stroke: A Translative Review from Bench to Bedside

**DOI:** 10.1155/2020/2979260

**Published:** 2020-08-28

**Authors:** Maciej Bladowski, Jakub Gawrys, Damian Gajecki, Ewa Szahidewicz-Krupska, Anna Sawicz-Bladowska, Adrian Doroszko

**Affiliations:** Department of Internal Medicine, Hypertension and Clinical Oncology, Faculty of Medicine, Wroclaw Medical University, Poland

## Abstract

Ischemic stroke remains the fifth cause of death, as reported worldwide annually. Endothelial dysfunction (ED) manifesting with lower nitric oxide (NO) bioavailability leads to increased vascular tone, inflammation, and platelet activation and remains among the major contributors to cardiovascular diseases (CVD). Moreover, temporal fluctuations in the NO bioavailability during ischemic stroke point to its key role in the cerebral blood flow (CBF) regulation, and some data suggest that they may be responsible for the maintenance of CBF within the ischemic penumbra in order to reduce infarct size. Several years ago, the inhibitory role of the platelet NO production on a thrombus formation has been discovered, which initiated the era of extensive studies on the platelet-derived nitric oxide (PDNO) as a platelet negative feedback regulator. Very recently, Radziwon-Balicka et al. discovered two subpopulations of human platelets, based on the expression of the endothelial nitric oxide synthase (eNOS-positive or eNOS-negative platelets, respectively). The e-NOS-negative ones fail to produce NO, which attenuates their cyclic guanosine monophosphate (cGMP) signaling pathway and—as result—promotes adhesion and aggregation while the e-NOS-positive ones limit thrombus formation. Asymmetric dimethylarginine (ADMA), a competitive NOS inhibitor, is an independent cardiovascular risk factor, and its expression alongside with the enzymes responsible for its synthesis and degradation was recently shown also in platelets. Overproduction of ADMA in this compartment may increase platelet activation and cause endothelial damage, additionally to that induced by its plasma pool. All the recent discoveries of diverse eNOS expression in platelets and its role in regulation of thrombus formation together with studies on the NOS inhibitors have opened a new chapter in translational medicine investigating the onset of acute cardiovascular events of ischemic origin. This translative review briefly summarizes the role of platelets and NO biotransformation in the pathogenesis and clinical course of ischemic stroke.

## 1. Ischemic Stroke: Its Burden and Classification

**Figure 1:**
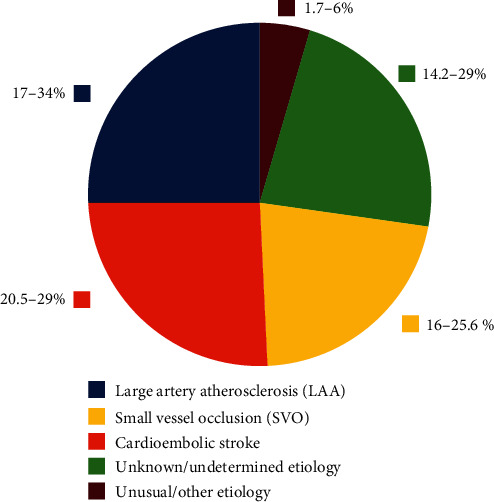
Incidence of each stroke subtype.

Cardiovascular disease (CVD) remains the main cause of morbidity and mortality, as reported worldwide annually. In spite of constant progress in diagnostic and therapeutic strategies, according to the recent data, there were estimated 72.72 million cases of CVD and 17.8 million CVD deaths in the world population. Stroke was the fifth cause of death globally with the morbidity reaching approximately 7.750 million and mortality 2.750 million in 2017 [1]. Ischemic stroke is the most common type of acute cerebrovascular event, responsible for 81% of all the stroke cases [2]. The thromboembolic event is a common denominator of all the subtypes of ischemic stroke. Large artery atherosclerosis (LAA) is the causative event in 17-34% of ischemic strokes and is characterized by activation of platelets along with thrombus formation on atherosclerotic plaque in extra/intracranial arteries (ruptured atherosclerotic plaque accompanied with a cascade of thromboinflammation). Small vessel occlusion/lacunar stroke (SVO) is diagnosed in 20.5-29.0% of cases, and it proceeds from lipohyalinosis (vessel wall thickening induced to the greatest extent by hypertension). Further ischemic stroke subtypes include cardioembolic (16-25.6%) which is predominantly generated by the atrial fibrillation (AF), then a stroke of unusual/other etiology (1.7-6%) and of unknown/undetermined etiology (14.2-29%) [3–6] (Figure 1).

Each of the noncardioembolic stroke subtypes is characterized by partially different pathophysiology, recurrence rate, magnitude of positive response to antiplatelet therapy, and survival rate (being relatively better for SVO than LAA strokes) [5, 7, 8]. Despite of the heterogeneous origin of particular subtypes of ischemic stroke, there are some uniform/common mechanisms, mostly related to increased activation of platelet-derived hemostasis. Hence, some common therapeutic strategies may reveal to be effective both in the treatment of an acute phase and in the primary and secondary stroke prevention.

## 2. Characteristics of the Cerebral Vascular Bed and Pathophysiology of Cerebral Ischemia-Reperfusion Injury

Cerebral arteries with their curvatures and bifurcations are characterized by a plaque-prone development anatomy. Contrary to the coronaries, carotid and cerebral vessels are subjected to high shear stress, which protects from atherosclerotic plaque enlargement but on the other hand also predisposes to intraplaque hemorrhage and plaque rupture [9]. Nevertheless, when hypercholesterolemia appears, endothelial dysfunction is promoted, limiting the positive action of physiological shear stress, and plaque formation is observed [10]. Some data suggest that low shear stress may change the expression of genes for inflammatory proteins leading to the origin of atherosclerosis-related inflammation [11, 12]. At high shear flow rates, as found in carotid/cerebral arteries or moderately stenosed vessels, the initial capture of circulating platelets to the endothelium is mediated by the von Willebrand factor (vWF) at the vascular wall without other stimulating factors [13]. It is suggested that the plasma level of vWF—to some extent—is a marker of endothelial cell damage and it predicts the onset and progression of atherosclerotic lesions in patients with hypertension. Hypotensive therapy, by non-drug-specific reducing endothelial damage and vWF expression, contributes to inhibition of both thrombus and atherosclerosis formation pointing thus at its protective role in the primary and secondary prevention of ischemic stroke [14].

## 3. Platelets as the Common Denominator of the Acute Ischemic Events and Pleiotropic Drug Target

Several years ago, Htun et al. have shown that patients with ischemic stroke or transient ischemic attack (TIA) were characterized by significantly increased P-selectin (CD62P) expression in platelets and circulating platelet-leukocyte aggregate concentration. Interestingly, other authors discovered that patients with the LAA infarction elicit higher platelet-leukocyte aggregate formation, when compared with the SVO group [15–17]. Differences in the CD62P concentration between stroke patients and controls returned to normal after 90 days of observation or gradually with implementation of antiplatelet treatment (stronger correlation with clopidogrel than with acetylsalicylic acid (ASA), but no association with warfarin treatment). Noteworthily, such treatment had no effect on normalization of the circulating platelet-leukocyte aggregate level in those patients [18, 19]. Initialization of thromboinflammation in ischemic stroke can be explained by the elevated platelet expression of the CD40 ligand (CD40L) in activated platelets, which, by triggering the expression of adhesive molecules, such as P-selectin, E-selectin, and ICAM-1, leads to formation of platelet-leukocyte aggregates [20, 21]. Moreover, Ishikawa et al. observed that, after induction of middle cerebral artery occlusion (MCAO) in the CD40-deficient rats, impaired platelet and leukocyte adhesion occurred leading to smaller brain infarct size in comparison to the control group [22]. Additionally, Jiang et al. demonstrated similar results in male rats treated with CD40 antagonist infusion before reperfusion of the occluded middle cerebral artery [23]. In human studies, high concentration of CD40 is associated with poor outcome at 3 months after ischemic stroke [15]. Moreover, leukocytes (especially regulatory T lymphocytes) have significant function in thromboinflammation during ischemic stroke by promoting ICAM-1 expression on platelets and endothelia which facilitates adhesion of granulocytes and platelets to the vessel wall [24, 25]. Hyperaggregable leukocytes, monocytes, and endothelia tend to activate platelets by platelet-activating factor secretion in the time of cerebral ischemia [26]. The PAF function is not only to activate adhesion of platelets and leukocytes (mostly neutrophils) to the damaged endothelium, but it also causes tissue edema through the increase of the vascular permeability in the peripheral tissues, increases secretion of granule-based enzymes in platelets, and enhances superoxide and arachidonate metabolism in neutrophils generating neurotoxicity leading to brain damage after ischemic stroke [27, 28].

Platelet secretion of thromboxane A2, adenosine diphosphate (ADP), matrix metalloproteinase-9 (MMP-9), and other platelet-derived soluble mediators promote thrombus formation in a positive feedback loop [29]. ADP is one of the most prevalent platelet activators under physiological condition. It also plays a significant role in cardiovascular disease development. Puurunen et al. in the prospective study of Framingham population identified platelet hyperreactivity to ADP to be associated with myocardial infarction and ischemic stroke incidence [30]. What is more, persistent elevation of platelet aggregation in response to ADP at three months after ischemic stroke is connected with more than threefold increased recurrence of stroke. Interestingly, cross-incubation of control platelets with plasma from stroke patients resulted in activation of platelets measured by the raised basal platelet calcium level and release of serotonin from platelets. These results, accompanied with the study by Dougherty et al. suggesting that ASA and dipyridamole treatment have no effect on platelet hyperreactivity to ADP, suggest that the lower threshold of platelet activation in ischemic stroke patients may be predominantly associated with the presence of plasmatic factors rather than with platelet functional disturbances [19, 31]. Recently, an increasing number of studies suggest that nitric oxide deficiency and nitric oxide synthase inhibitors can be one of the factors responsible for greater platelet aggregation in ischemic stroke patients.

## 4. The Role of Nitric Oxide Synthase and of Nitric Oxide in Ischemic Stroke

Endothelial vasodilative dysfunction, identified by decreased NO bioavailability, is a well-known risk factor for ischemic stroke. Changes in the nitric oxide concentration during the course of cerebral infarction can also be used as an important prognostic tool for ischemic stroke outcome. To date, three major isoforms of the NOS are described in the literature: neuronal constructive (nNOS), inducible (iNOS) and endothelial constitutive (eNOS). Each catalyzes the reaction of NO production, and in the catalytic cycle, the Fe^3+^+NO complex is the final intermediate from which in normal circumstances NO easily dissociates [32, 33]. However, nitric oxide overproduction autoinhibits the catalytic site of the NOS by reduction of iron to the stable Fe^2+^+NO complex [34]. In the presence of oxygen, the enzymatic inactive Fe^2+^+NO bond generates nitrate (reactive nitrogen species) and ferric ion, making the catalytic site of NOS again available for NO production. The described above oxygen dependency of NOS action plays a crucial negative role in ischemia and hypoperfusion [35]. Nitric oxide synthase produces not only NO and nitrates but also reactive oxygen species. Comparing to inducible NOS, eNOS and nNOS are responsible for higher production of superoxide, which is considered to be involved in atherosclerosis and recruitment of additional platelets to the sites of injury. On the other hand, iNOS and nNOS are more inclined than endothelial NOS to producing reactive nitrogen species (RNS) in which the undesirable role is to destabilize structure and function of proteins, leading to impaired catalytic activity of enzymes and even to cell apoptosis [36, 37].

**Figure 2:**
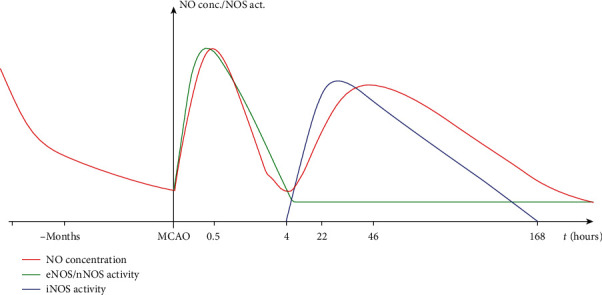
Concentration of nitric oxide and activation of NOS isoforms during the course of ischemic stroke.

After induction of middle cerebral artery occlusion, increased NO plasma concentration is observed for up to 30 minutes with its subsequent reduction in the following hours [38, 39]. After a gradual decrease, the level of NO and peroxynitrite (especially after reperfusion) increased again after 4 hours, reaching a maximum at 46 hours and lasting for up to seven days [40, 41]. The described above fluctuation of nitric oxide concentration is probably associated with different NOS subtype activities. The activity of eNOS and nNOS increases at the same time as nitric oxide concentration within the first minutes after induction of MCAO and significantly reduces thereafter [39]. The expression of iNOS is detected in the brain at 12-70 hours following cerebral ischemia and lasts up to 7 days, while the brain myeloperoxidase activity (a marker of neutrophil infiltration) is observed only after 4 hours, significantly increases at 22 h, and then decreases. These observations suggest that the initial increased level of NO after ischemia is connected with endothelial and neuronal nitric oxide production. While NO production by eNOS and nNOS slowly decreases, brain infiltration by neutrophils and their NO production by iNOS are responsible for the fluctuation of the NO bioavailability after ischemic stroke [40–42] Figure 2.

Dobrucki et al. observed lower concentration of NO before induction of ischemic stroke in spontaneously hypertensive rats (SHR) and higher concentration of O^2-^ release (connected with higher peroxynitrite production) after induction of middle cerebral artery occlusion leading to larger infarct size in SHR as compared to the control group [43]. Serrano-Ponz et al. found similar results in human studies. Those authors identified an increase in nitric oxide metabolites from day 1 to day 2 to be beneficial for the ischemic stroke patients as measured by the National Institutes of Health Stroke Scale (NIHSS) at day 7 and at 3 months and measured by the modified Rankin Scale at 3 months, while a steep increase of nitric oxide metabolite concentration from day 2 to day 7 was associated with a multiple increase in infarct volume [44]. According to Taffi et al., the high nitric oxide plasma level 30 days after cerebral infarct is associated with poor outcome in nonlacunar stroke, since a 10-unit increase in NO concentration predicts a 1-point reduction in the NIHSS score. Better outcome in patients with lacunar stroke is probably connected with higher concentration of NO in the first 24 hours after cerebral infarction and lower concentration of peroxynitrite [45].

At the molecular level and in animal model-based studies, during the first few hours after cerebral ischemia, nitric oxide production by eNOS is improving cerebral blood flow (CBF) within the ischemic penumbra (area of brain tissue surrounding the infarct that is at risk of infarction) in order to reduce infarct size and volume [46, 47]. It is documented that both eNOS-deficient mice and administration of eNOS inhibitors to rats provoke a decrease in absolute CBF in animals (up to 25–35% of the control level) [48, 49]. The activity of nNOS throughout 2 hours after reperfusion of MCAO is also enhanced. However, in nNOS-deficient mice, CBF is significantly higher after reperfusion, which suggests the adverse effect of nNOS activation during the course of ischemic stroke [50]. Zeng et al. found that hypoxic or ischemic brain injury during early reperfusion is associated with the generation of NO from nNOS which activates the early c-Jun N-terminal kinase 1/2—a signaling pathway involved in neuronal death [51]. Stagliano et al. proved that immediate administration of a specific inhibitor for nNOS (3-bromo-7-nitroindazole) after induction of common carotid artery thrombosis in rats accelerated sensorimotor recovery [52]. While some of the authors postulate that nonspecific inhibitors of eNOS and nNOS (L-N^G^-nitroarginine methyl ester (L-NAME)) reduce infarct size [53], others suggest that its biological function is partly dependent on simultaneous fluctuating N-methyl-D-aspartate (NMDA) concentration. NMDA in normal conditions is an activating neurotransmitter, but during ischemia, it is liberated from damaged neurons and has further neurotoxic activity. Globus et al. observed that brain lesions induced by NMDA was not affected by L-NAME administration. The reason for such a correlation is not fully understood. Activation of the NMDA receptor by neurotransmitters released from damaged neurons in the ischemic or penumbral area leads to NO overproduction and an increase in CBF in order to support the enhanced metabolic demand of the excited neurons. On the one hand, NO plays a role in the intracellular cascade of events leading to cell death following NMDA receptor activation; on the other hand, NO ensures adequate blood supply especially to the penumbral area. Probably, the final outcome of nitric oxide influence depends on the balance between these two processes [54, 55].

Nitric oxide produced by iNOS in the microglia (brain-based macrophages) may also lead to neuronal damage associated with the neurotoxicity mediated by NMDA receptors [56]. However, the main source of NO from iNOS during ischemic stroke originates from neutrophils. As mentioned before, infiltration of brain tissue by those phagocytes increases gradually during the first few days in the course of cerebral ischemia and reperfusion. Nitric oxide derived from neutrophils' iNOS is used in the inflammation process by peroxynitrite formation and in stimulation of neural apoptosis [57]. Garcia-Bonilla et al. discovered that after MCAO, iNOS-deficient mice engrafted with iNOS-positive bone marrow cells exhibited larger infarcts compared to iNOS-deficient mice autotransplanted with iNOS-deficient blood marrow cells. This study confirms that leukocytes play a significant role in the neuronal damage in ischemic stroke patients [58].

## 5. Pharmacological Approach: Nitric Oxide Donors and NOS Inhibitors

Some authors suggested that antiplatelet drugs affect nitric oxide biotransformation. According to Serebruany et al., modified-release dipyridamole and aspirin similarly increased primary diminished plasma eNOS activity in poststroke patients in comparison to the control group [59]. In Gelosa et al.'s study, ticagrelor given in the early phase after permanent MCAO in rats significantly attenuated chemotaxis of leukocytes and reduced expression of iNOS [60]. Zhao et al. showed no association between acetylsalicylic acid (ASA), clopidogrel, or dipyridamole administration and NO metabolites together with cyclic guanosine monophosphate (cGMP) levels in patients with prior ischemic stroke and in the control group [61].

Each NOS subtype plays different roles during ischemic stroke which is demonstrated in diverse effects observed by use of particular NOS inhibitors. Pretreatment with statins or Rho-kinase inhibitors improve cerebral blood flow in the ischemic area and penumbra, decrease cerebral infarct volume, and improve neurological function after MCAO by increasing eNOS activation.in mice [62, 63] Nevertheless, inhibition of eNOS by L-N-(1-iminoethyl)ornithine (L-NIO) before MCAO elevates iNOS expression and exacerbates brain damage [64]. Aminoguanidine (iNOS inhibitor) administration at 6 and 12 h after reperfusion in mice reduces NO concentration only in the penumbral region and lessens infarct size. Different iNOS inhibitors were studied by Armengou et al., in which N-(3-(aminomethyl)benzyl) acetamidine (1400W) administrated at the onset of ischemia and at 8-hour intervals for 3 days after MCAO resulted in a 55% reduction of infarct size, as measured 72 hours after induction of cerebral ischemia [65]. The protective role of iNOS inhibition in the thromboinflammation process is the most probably connected with decreased leukocyte activity. Matsuo et al. confirmed the essential role of neutrophils in ischemic stroke by documenting smaller infarct size in the neutropenic animals after reperfusion [66]. From a practical clinical perspective, edaravone, which directly enhances NO production, is recommended by Japanese guidelines for neuroprotection in ischemic stroke patients within 24 hours of onset [67]. This drug is commonly used in amyotrophic lateral sclerosis and exerts also a neuroprotective effect in reperfusion injury by reducing levels of superoxide increasing NO production and decreasing nNOS expression in cerebral neurons [68]. According to the Acute Infarction Study Group, administration of this substance < 72 h after ischemic stroke and through 14 days was connected with significant improvement in functional outcome evaluated by the modified Rankin Scale [69]. What is more, in Feng et al.'s systemic review on the edaravone influence on patients with acute ischemic stroke, the use of the drug was associated with neurological improvement in the intervention group compared with the control group (RR = 1.99) [70].

The plasma level of L-arginine (a substrate for NOS) is decreased in patients following the ischemic stroke and subsequently rises between 6 and 24 hours after the event. According to Armengou et al., plasma L-arginine concentrations are negatively correlated with the infarct volume and are significantly lower in patients with early neurologic deterioration as well as in those with poor outcome [65]. In Morikawa et al.'s study, administration of L-arginine 5 minutes after MCAO reduced infarct volume in rats measured 24 hours after vessel occlusion [47]. Lower levels of free radical production leading to smaller infarct size were also documented in Mason et al.'s and Zhao et al.'s animal studies of direct nitric oxide donor (diethanolamine nitric oxide and ZJM-289 novel NO-releasing derivative of 3-n-butylphthalide) administration during reperfusion [71, 72]. Although in human studies the transdermal glyceryl trinitrate (GTN) administration < 48 h and <5 days after cerebral ischemia did not improve functional outcome for ischemic stroke patients, in Woodhouse et al.'s analysis, transdermal GTN was safe and correlated with better functional outcome and with fewer deaths when administered within 6 hours of stroke onset. Significant beneficial effects were also achieved in disability (Barthel Index), quality of life, cognition, and mood [73–75]. Willmot et al. in their meta-analysis of preclinical studies confirmed the time-dependent effect of NO donors and L-arginine administration, specifying that early treatment (within 60 minutes) of ischemia was associated with the highest outcomes in comparison to neutral ones in those studies assessing treatment up to 48 hours following induction of ischemic stroke [76].

In human cells, NOS converts L-arginine to L-citrulline with a concomitant synthesis of NO, while asymmetric dimethylarginine (ADMA) is the most potent competitive inhibitor of this reaction. ADMA could play a crucial role in the CVD development as its higher plasma concentrations are significantly associated with cardiovascular risk factors, such as intima-media thickness of the carotid artery [77], hypertension [78], and diabetes mellitus types 1 and 2 [79, 80]. What is more, Ercan et al. showed that the plasma ADMA level measured during the first 24 hours in the group of patients after acute ischemic stroke was significantly higher than that in the control group [81]. Petrova et al. documented earlier reduction of plasma ADMA concentration in stroke patients after thrombolysis in comparison to the no-reperfusion group [82]. On the other hand, only symmetric dimethylarginine (with no effect on NO production) was a predictor of mortality in patients after acute ischemic stroke during 7.4 years of follow-up, while no correlation for ADMA was noted [83]. It seems that the intracellular compartment could be more significant in pathophysiology of CVD than its plasma level. Masuda et al. found the endothelial concentration of ADMA to be up to 10-fold higher than in plasma, while Yokoro et al. showed that protein arginine N-methyltransferase 1 (PRMT1) and dimethylarginine dimethylaminohydrolase-1 (DDAH-1)—enzymes responsible for the biotransformation of ADMA—are expressed also in erythrocytes, leukocytes, and platelets. Those authors also suggested that the side effect of protein methylation (a protective mechanism against highly reactive oxygen-derived free radicals) can lead to ADMA overproduction, which in consequence lowers cellular NO production, can cause endothelial damage, and can increase platelet activation and aggregation [84–87]. However, to date, there is no study conducted analyzing the association between nitric oxide biotransformation (including ADMA, PRMT1, and DDAH-1) and human platelets of ischemic stroke patients.

## 6. Platelet-Derived Nitric Oxide (PDNO)

A vast majority of studies documented the association between decreased endothelial NOS expression and clinical disorders predisposing to stroke, such as diabetes mellitus, atherosclerosis, hypertension, and cigarette smoking in patients [88–91]. Radomski et al. were the first authors to describe the inhibitory role of platelets' NO production on a thrombus formation. It has been found that L-arginine administration increases platelet NO formation leading to cGMP synthesis and protein kinase G (PKG) activation in thrombocytes' cytosol which in consequence inhibits thrombus formation. The described NO/cGMP/PKG pathway's antiaggregatory properties in platelets depend on provoking Ca^2+^ sequestration and inhibiting platelet degranulation [92] by

1. refilling intraplatelet Ca^2+^ stores by promoting sarcoplasmic reticulum adenosine triphosphatase (ATPase), decreasing intracellular Ca^2+^ levels, and inhibiting influx of Ca^2+^ [93]
2. inhibition of the inositol 1,4,5-trisphosphate-stimulated Ca^2+^ release from the sarcoplasmic reticulum [94]
3. attenuating the TxA_2_ receptor function by its phosphorylation [95]
4. phosphorylation of vasodilator-stimulated phosphoprotein (VASP), which enables VASP binding to the platelet cytoskeleton leading to inhibition of proaggregatory glycoprotein IIb/IIIa (GPIIb/IIIa) activation [96]

Without PKG action, cGMP also prevents platelet activation by inhibition of phosphodiesterase type 3 which increases intracellular cyclic adenosine monophosphate (cAMP; potent antiaggregation factor) [97] and inhibits phosphoinositide 3-kinase leading to GPIIb/IIIa fibrinogen receptor inactivation [98]. Nitric oxide blocks thrombus formation also on cGMP-independent mechanisms. The nitrosylation of the N-ethylmaleimide-sensitive factor inhibits aggregation by downregulating alpha granule secretion and GPIIb/IIIa activation [99, 100]. What is more, irreversible nitration of platelet proteins by peroxynitrites results in inhibition of platelet adhesion to fibrinogen and decreased aggregation [101, 102].

Moreover, many other authors also showed that upon activation, platelets produce NO which inhibits adhesion and aggregation [92, 103–106]. Williams et al. demonstrated that high shear stress alone is sufficient to increase NO production in platelets leading to reduction of thrombus generation under blood flow. Moreover, authors described that reduction in thrombus formation (at a shear rate of 1000 s^−1^) was abolished in the presence of L-NAME (the NO inhibitor), while at venous levels of shear rate (100 s^−1^), this substance had no effect on platelet activation and aggregation [107]. Cozzi et al. showed that platelet deposition is inversely related to platelet NO production and that intracytoplasmic Ca^2+^ elevation triggers platelet NO formation. Those results can suggest that the increase in intraplatelet Ca^2+^ concentration enhances the NO production which, in turn, limits thrombus size [108].

Some of the authors suggest that the generation of nitric oxide by resting platelets is constant (and is not elevated by L-arginine administration) [92, 103, 109]. Li et al. showed that basal production of NO by platelets activates cGMP-dependent protein kinase G (PKG) and enhances vWF-induced activation of platelets, which promotes rather than inhibits thrombus formation [110]. Those results can lead to the conclusion that platelet responses to NO and cGMP are both pro- and antiaggregative. However, in a study by Radziwon-Balicka et al., incubation with L-arginine inhibited platelet aggregation (by generation of NO) regardless of the platelet-activating stimulus concentration [111]. As a result from these studies, nitric oxide may serve as a platelet negative feedback regulator alone and only additional reaction of nitric oxide with superoxide anion can promote enhanced thrombus formation [112, 113].

## 7. Expression of the Nitric Oxide Synthase in Platelets

For many years, there was controversy whether platelets express their own nitric oxide synthase producing PDNO. Some of the authors suggested that contamination platelet samples by leukocytes account for suspected platelet NO production [114]. However, in a study on platelet subpopulations, Radziwon-Balicka et al. achieved high purity of their isolations (<2 leukocytes/100 000 platelets) and still detected significant nitric oxide production. Salvemini and colleagues showed that leukocyte contamination of >1% can inhibit aggregation via a NO-dependent mechanism, while in Radziwon-Balicka et al.'s study, leukocyte contamination was less than 0.002%. Finally, the authors concluded that this low leukocyte concentration in samples cannot account for detected NO production in the analyzed probes [112, 115–117].

In human megakaryocytes, both endothelial and inducible nitric oxide synthase isoforms are detected [118]. But whether platelets have the capacity to synthesize iNOS remains uncertain [119]. There is an interesting hypothesis that iNOS detected in platelet sample could derivate from leukocyte contamination. However, in Radziwon-Balicka et al.'s study, in the leukocyte-free probe samples (<2 leukocytes/100 000 platelets) the iNOS-selective antagonist 1400W was unable to reverse the antiaggregating effect of L-arginine. According to this study, the presence of iNOS in platelets is improbable [112].

Some authors also postulated that platelets do not contain eNOS and that this NOS isoform exists only in endothelial cells. However, Radziwon-Balicka et al. ultimately confirmed the presence of eNOS in triton-resistant platelet caveolae by a more specific identification method (fluorescence-activated cell sorting, while others used mass spectrometry) [112, 114, 120]. Both in animal and human studies, the endothelial nitric oxide synthase isoform is proved to produce PDNO [112, 121, 122]. Freedman et al. demonstrated that bleeding time was significantly decreased in eNOS-deficient versus wild-type mice. What is more, the bleeding time in thrombocytopenic eNOS-deficient mice transfused with eNOS-deficient platelets was significantly decreased compared with the same breed of mice transfused with wild-type platelets [121]. Moreover, Morrell et al. showed that the infusion of platelets from eNOS-deficient mice to animals with normal expression of eNOS resulted in increased granule exocytosis and stimulation of aggregation [99]. Riba et al. documented that vWF connection with platelet Gp Ib not only stimulates adhesion and aggregation but also activates platelet eNOS (measured by the increase in cGMP formation) with the presence of ADP and TxA_2_. Interaction between collagen and platelet glycoprotein VI (GPVI) receptor activated platelet eNOS (with costimulation by ADP and TXA_2_) only partially [123, 124]. Freedman et al. in a different study showed that inhibition of platelet eNOS increased P-selectin expression on the platelet surface after stimulation with ADP [106]. P-selectin is essential for leukocyte-platelet complex formation, and inhibition of platelet eNOS enhances the formation of those aggregates (especially monocyte-platelet aggregates) [125, 126].

## 8. Subpopulations of Platelets

Initially, the diversity among platelet size and density was attributed to the platelet aging processes. The large-dense platelets were identified as young thrombocytes recently released into the streaming blood, whereas the small and low-density ones were postulated to represent older subpopulation. Nevertheless, studies by the Thompson et al. and Penington et al. have demonstrated that platelet size heterogeneity depends rather on platelet production from the different three ploidy classes of megakaryocytes (differ in their organelle content concentration) [127, 128]. Large-dense platelets contain a greater amount of glycogen, orthophosphate, and ADP and are characterized by upregulated glycolysis, glycogenolysis, and protein synthesis than small and low-density ones [129]. Large-dense thrombocytes aggregate more (due to higher ADP release and lower ADPase activity), require higher amount of prostacyclin concentration to inhibit aggregation, and adhere stronger to collagen (due to higher expression of the membrane GPIa/IIa receptors) than small and low-density ones [130–132]. Simultaneously, other authors showed that small and low-density platelets have an enhanced intracellular Ca^2+^ response to thrombin, which provokes them to a greater aggregation in response to these stimuli. Moreover, small and low-density platelets comparing to large-dense ones contain lower levels of the phosphorylated form of vasodilator-stimulated proteins, which can be the consequence of their weaker response to antiaggregative NO stimulation [133–135]. Although large-dense thrombocytes have higher aggregation and adhesion ability, small and low-density platelets react greater on thrombogenic stimuli with lower autoinhibitory response to NO.

In Kiliçli-Camur et al.'s study, high mean platelet volume (MPV) (associated with platelets' large and dense subpopulation) was increased during acute myocardial infarction and in the first subsequent weeks. What is more, patients with coronary artery disease (CAD) and elevated MPV had greater risk of acute myocardial infarct in comparison to those with a lower MPV, regardless of the extent of the coronary lesions [136]. Many studies also connected high MPV with ischemic stroke. In Butterworth and Bath's study, MPV was significantly higher in the ischemic stroke group than in the controls. Additionally, in stroke subgroup analysis, MPV was associated with cortical stroke but not with lacunar stroke [137]. Moreover, in Özkan et al.'s study, high MPV was associated with acute ischemic stroke only in patients with noncardioembolic stroke (with sinus rhythm and without heart failure or left atrial enlargement) [138]. More importantly, high MPV predicts also the risk of a second stroke up to 4 years before the acute event (11% increase of the relative risk of stroke for each femtoliter of MPV increase) and unfavorable outcome after cerebral infarction (death or dependency at 3 months follow-up) [137, 139]. These results underline the influence of platelets in CVD development and additionally suggest that MPV could be another risk factor for CVD development and progression.

## 9. Clinical Importance of PDNO and Expression of eNOS in Platelet Subpopulation

Radziwon-Balicka et al. identified in humans the thrombocyte subpopulations based on the presence of endothelial nitric oxide synthase (eNOS-positive or eNOS-negative platelets). Thrombocytes that are eNOS-negative constitute about 20% of total human platelet population and fail to produce NO, which attenuates their cGMP signaling pathway and—as result—promotes adhesion and formation of larger aggregates. The authors postulate that the role of e-NOS-negative platelets in thrombogenesis is probably to initiate adhesion and aggregation (the seed platelet hypothesis), while e-NOS-positive ones limit thrombus formation through NO production [112]. In the presence of vascular injury, eNOS-negative thrombocytes are the first to adhere to exposed collagen and/or to the von Willebrand factor. Thanks to the absence of endogenous NO generation, a quicker activation of integrin *α*IIb*β*3 appears, alongside with stabilization of initial rolling and adhesion of platelets [140]. Further recruitment of eNOS-positive platelets to a site of injury and formation of a greater aggregate is supported by matrix metalloproteinase secreted by eNOS-negative thrombocytes. Following aggregation, the eNOS-positive platelets form the bulk of a thrombus due to their greater thromboxane generation in comparison to the eNOS-negative thrombocytes. Finally, the limitation of aggregate size is achieved through nitric oxide generation by eNOS-positive platelets, when their number in the thrombus overbalances eNOS-negative ones [112].

Some data suggest that platelet-derived nitric oxide (PDNO) might be connected with development of cardiovascular disorders, including ischemic stroke [91, 141]. Ikeda et al. showed a negative correlation between PDNO and age, mean arterial pressure, total cholesterol, and LDL-cholesterol level. What is more, the PDNO release was also significantly decreased in long-term smokers [142]. Queen et al. has demonstrated that platelet nitric oxide synthase activity at baseline was lower in diabetic patients than in control subjects, while the platelet nitric oxide generation stimulated by beta-adrenoceptors attenuated in the course of diabetes [143]. Another study postulates that lower PDNO production was an independent predictor for acute coronary syndrome with odds ratio reaching 4.0 [144]. Laufs et al. showed the influence of PDNO on the course of ischemic stroke pointing out that statin-related improvement in the outcome is mediated by the increase in the eNOS expression in the thrombocytes and aorta [145]. Therefore, changes in the eNOS-negative to eNOS-positive platelet ratio might result in modification of the risk and outcome of acute ischemic cardiovascular events such as ischemic stroke or acute coronary syndrome [112].

On the other hand, there is still some controversy about association between nitric oxide and ischemic stroke. Platelets are characterized by the expression of several activation pathways. Noteworthily, Taka et al. showed that the NO donor and the NO synthase inhibitor did not affect shear-induced platelet reactivity or vasodilatation in stroke-prone spontaneously hypertensive rats [146]. Interestingly, Lafrati et al. found that eNOS-deficient animals showed a prolongation of time to occlusion, which was explained by the compensatory mechanism. Although eNOS-deficient mice had increased platelet recruitment, simultaneously they had also enhanced fibrinolysis due to lack of NO-dependent inhibition of Weibel-Palade body release (containing tissue plasminogen activator) from the endothelium [147]. What is more, results from other studies show that a cumulative effect of NO on ischemic stroke could cause harm; as in animals treated at reperfusion with the nonselective NOS inhibitor, the infarct volume was significantly almost twofold decreased [148]. Manickam et al. suggests also that inhibition of peroxynitrite and other ROS production by superoxide dismutase rather than nitric oxide itself protects against ischemia/reperfusion injury in the brain [149]. Hence, the Janus-faced action of NO in stroke requires further precise studies.

## 10. Prevention and Treatment of Ischemic Noncardioembolic Stroke: A Translational Focus on Platelets in the Shade of Current Guidelines and Trials in Cardiovascular Medicine and Neurology from Bench to Bedside

Specific therapeutic strategy for ischemic stroke is thrombolytic therapy (alteplase treatment), in which efficacy has been clearly shown especially when performed within the therapeutic time frame (up to 4.5 hours from the onset of stroke symptoms and in particular cases, if the risk-benefit ratio approves its implementation, within 6 hours) [150]. Additionally, every patient eligible for mechanical thrombectomy (complementary treatment option to alteplase infusion) should have previous thrombolysis performed (depending on inclusion/exclusion criteria). According to the American Heart Association/American Stroke Association (ASA/AHA) 2019 guidelines, intravenous aspirin should not be administered within 90 minutes after the start of i.v. alteplase treatment because it increases risk of symptomatic intracranial hemorrhage more than twofold without any positive effect on functional outcome within 3 months of observation. The safety and efficacy of i.v. glycoprotein IIb/IIIa inhibitors administered after alteplase infusion or thrombectomy is uncertain [151–154].

Intravenous administration of tirofiban is the most commonly used antiplatelet therapy following rescue angioplasty with or without stenting after myocardial infarction. In acute ischemic stroke, tirofiban has been reported to facilitate further recanalization if primary mechanical thrombectomy failed and the highest benefit was achieved in LAA ischemic stroke subtype. Thus, tirofiban can be an interesting adjuvant therapy after unsuccessful thrombolysis/thrombectomy [155–157]. However, recent guidelines for the early management of patients with acute ischemic stroke recommends consideration of antiplatelet/antithrombotic therapy < 24 hours after treatment with i.v. alteplase only if the patient has concomitant conditions for which such treatment given in the absence of i.v. alteplase is known to provide substantial benefit or withholding such treatment is known to cause substantial risk. This recommendation is based only on a single-center retrospective analysis, which found no increased risk of hemorrhage with early initiation of antiplatelet or anticoagulant therapy after i.v. alteplase or endovascular treatment compared with initiation > 24 hours after ischemic stroke [154, 158]. Those recommendations prevent clinicians from wide usage of tirofiban in ischemic stroke patients after unsuccessful thrombolysis.

Approximately 8% of patients with ischemic stroke are admitted to the hospital in the time window allowing thrombolysis procedure [159]. For the remaining, about 90% of the only available current treatment option is secondary prevention of ischemic stroke. According to AHA/ASA 2019 guidelines for stroke prevention, administration of acetylsalicylic acid (160-300 mg/24 h) is recommended in patients with acute ischemic stroke within 24 to 48 hours after onset of disease or >24 hours after alteplase treatment with lifetime continuation of such antiplatelet treatment [154]. In Chen et al.'s meta-analysis, early use of ASA (<48 h) in acute ischemic stroke decreased the risk of recurrent stroke or death in a hospital with a nonsignificant increase in hemorrhagic stroke or hemorrhagic transformation of the original infarct [160]. In animal studies, the high-dose ASA therapy in temporary induced ischemia significantly reduced infarct size compared to placebo, in humans corresponding dosage would account for 19 grams of ASA with probably unfavorable benefit/risk ratio (higher hemorrhage risk) [161]. What is more, patients with diagnosed minor noncardioembolic ischemic stroke (NIHSS score ≤ 3) or at high-risk transient ischemic attack (TIA) (ABCD_2_ (Age, Blood Pressure, Clinical Features, Duration, Diabetes) score ≥ 4) who did not receive thrombolysis should be treated with dual antiplatelet therapy (ASA and clopidogrel) started within 24 hours after symptom onset and continued for 21 days [162]. Finally, ASA alone is significantly reducing the 6-week risk of recurrent ischemic stroke by about 60% and disabling or fatal ischemic stroke by about 70% (with the greatest benefit in patients with TIA or minor stroke) [163]. However, according to the CAST study, the number needed to treat for ASA to prevent one stroke within one year is 100 patients [164].

Acetylsalicylic acid is a drug of choice in the secondary prevention of ischemic stroke. In case of intolerance, it can be replaced by clopidogrel 75 mg daily according to 2017 ESC Guidelines on the Diagnosis and Treatment of Peripheral Arterial Diseases [165, 166]. The meta-analysis by Paciaroni et al. even postulates clopidogrel to be a better choice in the secondary prevention of ischemic stroke due to the significant risk reduction for major adverse cardiovascular and cerebrovascular events, any ischemic or hemorrhagic stroke, and recurrent ischemic stroke in patients who received clopidogrel versus ASA. The risk of bleeding was also lower for clopidogrel in comparison to acetylsalicylic acid [167]. However, 2019 updated guidelines for the early management of patients with acute ischemic stroke suggest that increasing the dose of acetylsalicylic acid or switching to an alternative antiplatelet agent in patients who have a noncardioembolic ischemic stroke while taking ASA is still not well established [154]. There are only few indications for dual ASA and clopidogrel therapy mainly due to high risk of life-threatening hemorrhages. Dual antiplatelet therapy is indicated in minor noncardioembolic ischemic stroke or with high-risk TIA (as described before), after myocardial infarct or after carotid artery stenting [154, 166, 168]. The benefit of single antiplatelet therapy for preventing stroke in asymptomatic patients with carotid artery stenosis > 50% is not proven to be beneficial in randomized control trials. However, optimal medical treatment with acetylsalicylic acid or clopidogrel is recommended for the majority of those patients in the primary prevention of ischemic stroke to reduce the risk of stroke and other cardiovascular events, as these patients are also at twice the risk for myocardial infarct. In symptomatic extracranial carotid stenosis, antiplatelet monotherapy is always recommended [166]. Newer drugs from the same class as clopidogrel (inhibitors of P2Y12 receptor: prasugrel, ticagrelor, and cangrelor) are not beneficial in the acute phase of ischemic stroke. Vorapaxar, protease-activated receptor-1 antagonist (PAR-1), treatment during acute myocardial infarct is proved to be beneficial in clinical tests; its use in acute ischemic stroke is harmful leading to a greater hemorrhagic transformation [154, 169]. Another antiplatelet drug, abciximab (glycoprotein IIb/IIIa inhibitor) as medical treatment for the secondary prevention of ischemic stroke, is potentially harmful and should not be used, while efficacy of eptifibatide is not well established yet [154, 170].

Even though the main function of antiplatelet agents is to inhibit platelet-platelet aggregation, in case of penumbral protection, platelet-endothelium adhesion and platelet-leukocyte aggregation are similarly important. Anfibatide by inhibiting adhesive properties of platelets (blocker of platelet glycoprotein receptor Ib) significantly reduces infarct size, increases the number of intact neuronal cells, and improves neurobehavioral function by reducing postischemic blood-brain barrier damage, leukocyte migration, and microthrombus formation [171, 172]. Caplacizumab (humanized anti-vWF antibody) by binding to vWF inhibits platelet adhesion to the vessel wall (platelet GPIb—vascular vWF interaction blockade). This drug in Momi et al.'s study both prevented middle carotid artery thrombosis and reduced brain damage without provoking hemorrhage by inducing reperfusion when given before or up to 15 minutes after complete artery occlusion. Tirofiban (GPIIb/IIIa blocker) prevented thrombosis but did not induce reperfusion and caused striking brain hemorrhage [173, 174]. The activity of vWF is regulated by a disintegrin-like and metalloprotease with thrombospondin type I repeats-13 (ADAMTS13) that transforms vWF to smaller, less active forms. According to Zhao et al., infusion of a high dose of recombinant human ADAMTS13 into a wild-type mouse immediately before reperfusion reduces infarct size and improves functional outcome without producing cerebral hemorrhage pointing thus at ADAMTS13 to be a useful potential therapeutic target in ischemic stroke [175]. Another potential drug connected with GPIb interactions is specific factor XIIa inhibitor rHA-infestin-4. This substance completely inhibits occlusive arterial thrombus formation in mice and rats while leaving hemostasis fully intact [176]. The revacept (GPVI-Fc fusion protein) blocks competitively binding of vWF to collagen and GPVI-mediated platelet adhesion. Goebel et al. showed that this medication prevents thrombus formation after endothelial injury and, if applied immediately before reperfusion in mice with ischemic stroke, significantly improves functional outcome and decreases cerebral infarct size [177]. de Brito Toscano et al. showed that a platelet-activating factor (PAF) receptor-deficient mouse had a smaller brain-infarcted area in comparison to the control group [178]. Furthermore, pretreatment with F-0401, dihydropyridine calcium antagonist with PAF antagonistic action, prevents the occurrence of brain edema, disruption of the blood-brain barrier, and neuronal damage caused by cerebral ischemia [179]. Pretreatment with PAF antagonist (BN 50739) before induction of focal cortical lesions in anesthetized rats improved the penumbral cerebral blood flow and reduced edema and the progression of neuronal damage [180]. Inclacumab (a potent and selective P-selectin-neutralizing antibody) appears to reduce myocardial damage after percutaneous coronary intervention (PCI) in patients with non-ST-segment elevation myocardial infarction. However, there is no study conducted analyzing effects of inclacumab on ischemic stroke [181] (Table 1).

Despite the numerous antiplatelet drugs implemented in clinical practice and large body of evidence for their effectiveness in particular clinical scenarios, the precise and disease-dedicated therapy remains a difficult task for clinicians. Further studies, based more and more on translational medicine, are required to combine pathophysiological knowledge and the basics of pharmacotherapy with data from epidemiological clinical trials in order to formulate optimal recommendations.

## 11. Clinical Implications and Future Directions

In summary, there is a large body of evidence on the important role of nitric oxide in the pathophysiology of thrombogenesis in patients at high and very high cardiovascular risk. However, there are scarcely no studies separating the contribution of endothelial and platelet-derived NO in the onset and clinical course of particular cardiovascular events of atherothrombotic origin. The study discovering the eNOS-negative and eNOS-positive subpopulations of platelets constitutes a milestone that may change the paradigm stating that decreased endothelial NO bioavailability and endothelial dysfunction itself may promote the onset of acute ischemic events. What is more, the quantitative analysis accompanied with the verification of some activation-related platelet features might become a useful tool as a prognostic biomarker for ischemic stroke and thromboinflammation. Furthermore, the role of ADMA in platelets, which elevated the plasma level, is a well-known cardiovascular risk factor and requires further studies. A detailed analysis of the platelet ADMA biotransformation (including its synthesis with PRMT, transmembrane translocation with CAT, and degradation by the DDAH) should provide some new important data on this issue. Future experimental studies using the selective iNOS and nNOS inhibitors or antiplatelet agents blocking the GPI and GPVI receptors in the management of brain ischemia-reperfusion injury are required to clarify the Janus-faced action of nitric oxide in stroke.

## Figures and Tables

**Table 1:** Effect of different antiplatelet drug treatments on outcome in ischemic stroke.

	Mechanism of action	Primary prevention of ischemic stroke		Acute phase of ischemic stroke		Secondary prevention of ischemic stroke	
		Animal studies	Human studies	Animal studies	Human studies	Animal studies	Human studies
Acetylsalicylic acid	Cyclooxygenase inhibitor	Beneficial [182]	Neutral (beneficial after artery stenting) [166]	Beneficial [161]	Beneficial in TIA and minor stroke (NIHSS ≤ 3) [183]	Beneficial [184]	Beneficial [154]
Clopidogrel	Inhibitor of P2Y12 receptor	Beneficial [185]	Neutral (beneficial after artery stenting) [166]	Beneficial [186]	Beneficial in TIA and minor stroke (NIHSS ≤ 3) [183]	Beneficial [185]	Beneficial [154]
Prasugrel	Inhibitor of P2Y12 receptor	Beneficial [187]	Neutral (beneficial after ACS) [188]	Beneficial [186]	Harmful [154]	Beneficial [189]	Harmful [190]
Ticagrelor	Inhibitor of P2Y12 receptor	Beneficial [191]	Neutral (better prevention with higher hemorrhage incidence) [192]	Beneficial [191]	Harmful [154]	No data found	Neutral (better prevention with higher hemorrhage incidence) [193]
Cangrelor	Inhibitor of P2Y12 receptor	Neutral [188]	No data found	Beneficial [186]	Harmful [154]	No data found	Beneficial in stroke prevention in the perioperative period [194]
Vorapaxar	PAR-1 antagonist	No data found	Harmful [195]	No data found	Harmful [169]	No data found	Harmful/neutral [196, 197]
Tirofiban	GPIIb/IIIa blocker	Beneficial (group effect) [198]	No data found [199] (neutral/harmful in the second-generation GPIIb/IIIa blockers)	Beneficial [172]	Beneficial [157]	Beneficial [198]	Uncertain [200]
Abciximab	GPIIb/IIIa blocker	Beneficial (group effect) [198]	No data found [199] (neutral/harmful in the second-generation GPIIb/IIIa blockers)	Beneficial [201]	Uncertain [154]	Beneficial [198]	Harmful [199]
Eptifibatide	GPIIb/IIIa blocker	Beneficial (group effect) [198]	No data found [199] (neutral/harmful in the second-generation GPIIb/IIIa blockers)	Beneficial [201]	Beneficial [151]	Beneficial [198]	Uncertain [200]
Anfibatide	GPIb blocker	Beneficial [202]	No data found	Beneficial [172]	No data found	No data found	No data found
Caplacizumab	Anti-vWF antibody, blocker of platelet GPI-vWF adhesion	Beneficial [173]	No data found	Beneficial [173]	No data found	No data found	No data found
ADAMTS13	Recombinant human enzyme transforming vWF to smaller, less active forms	Beneficial [175]	No data found	Beneficial [203]	No data found	No data found	No data found
rHA-infestin-4	XIIa inhibitor	Beneficial [176]	No data found	Beneficial [204]	No data found	No data found	No data found
Revacept	Competitive blocker of platelet GPVI adhesion to vWF	Beneficial [177]	Beneficial [205]	Beneficial [206]	No data available (ongoing study of patients with stable coronary artery disease undergoing elective PCI)	No data found	No data found
F-0401	Dihydropyridine calcium antagonist with PAF antagonistic action	Beneficial [179]	No data found	Beneficial [207]	Beneficial (in the study of human astrocytoma and neuroblastoma cells) [207]	No data found	No data found
BN 50739	PAF antagonist	Beneficial [180]	No data found	Beneficial [209]	No data found	No data found	No data found
Inclacumab	P-selectin neutralizing antibody	No data found	No data found	No data found	No data available (beneficial in non-ST-segment elevation myocardial infarction) [181]	No data found	No data found
